# Association between fat-to-muscle ratio and diabetic kidney disease: a nationwide NHANES analysis with real-world validation

**DOI:** 10.3389/fnut.2025.1700718

**Published:** 2025-11-28

**Authors:** Xiaohong Zhang, Jiaqun Lin, Wenfeng Wang, Zishan Lin, Yuan Fang, Yanfang Xu, Jianxin Wan

**Affiliations:** 1 Department of Nephrology, Blood Purification Research Center, the First Affiliated Hospital, Fujian Medical University, Fuzhou, China; 2 Research Center for Metabolic Chronic Kidney Disease, the First Affiliated Hospital, Fujian Medical University, Fuzhou, China; 3 Department of Nephrology, National Regional Medical Center, Binhai Campus of the First Affiliated Hospital, Fujian Medical University, Fuzhou, China

**Keywords:** fat-to-muscle mass ratio, diabetic kidney disease, NHANES, real-world validation, risk factor

## Abstract

**Background:**

Obesity and abnormal body composition are recognized contributors to diabetic kidney disease (DKD) development. The fat-to-muscle mass ratio (FMR), an indicator of body composition, remains insufficiently studied in relation to DKD risk.

**Methods:**

This study was a nationwide cohort analysis utilizing data from eight National Health and Nutrition Examination Survey (NHANES) cycles. FMR was derived using dual-energy X-ray absorptiometry (DXA) and evaluated in both categorical and continuous forms. Given the cross-sectional design of NHANES for DKD status assessment, the association between FMR and DKD was analyzed as a prevalence association. Mortality outcomes were further evaluated via retrospective linkage to the National Death Index, forming a retrospective mortality cohort among prevalent DKD cases. To validate the association between FMR and DKD prevalence, we additionally analyzed an independent hospital-based clinical cohort, in which FMR indices were also measured by DXA, and a logistic regression analysis was performed.

**Results:**

After applying the exclusion criteria, 680 DKD patients were included in the analysis. Over a median follow-up of 97 months, 267 deaths (37.58%) were recorded. DKD patients exhibited significantly higher arm-FMR, trunk-FMR, and total-FMR values. A logistic regression analysis revealed that arm-FMR, trunk-FMR, and total-FMR were independently associated with an increased DKD risk (all *p* < 0.0001). Stratified subgroup analyses further confirmed significant associations between FMR and DKD, with notable interactions observed in arm-FMR and trunk-FMR when stratified by age and sex. The receiver operating characteristic curve analysis demonstrated that trunk-FMR exhibited the strongest predictive value for DKD (AUC = 0.812, sensitivity = 85.9%, specificity = 63.8%). The Kaplan–Meier survival curves revealed that lower FMR quartiles were associated with better survival outcomes for both all-cause and CVD mortality among DKD patients (all log-rank *p* < 0.001). Moreover, non-linear associations were detected between FMR and DKD prevalence, as well as between FMR and mortality outcomes. In the real-world validation cohort consisting of 94 patients, a univariate logistic analysis revealed that all FMRs were identified as risk factors for the development of DKD. Another multivariate logistic analysis revealed that trunk-FMR exhibited the highest predictive model value (OR = 12.029, 95% CI 1.431–121.317, *p* = 0.026, AUC = 0.735).

**Conclusion:**

This NHANES-based study identified a robust association between FMR and DKD prevalence, along with all-cause and CVD mortality. Importantly, these associations were further supported by an independent real-world clinical cohort, underscoring the robustness and generalizability of our findings. Optimizing FMR may play a pivotal role in improving the prognosis of DKD patients.

## Introduction

Diabetes mellitus (DM) presents a critical public health issue, with a rising prevalence worldwide (1). Projections indicate that, by 2030, approximately 643 million individuals will be affected by diabetes (2). Among them, 35–40% may develop diabetic kidney disease (DKD), a progressive disorder that substantially increases the risk of end-stage kidney disease and mortality (3). Given its high prevalence and severe outcomes, DKD has become a major global health concern.

Obesity and dysregulated body composition are key drivers of the development and advancement of DM and its associated complications (4). The interplay between obesity and type 2 DM is influenced by both environmental factors and genetic predispositions (5). Traditionally, BMI, a widely used metric, served as a standard measure of obesity; however, it fails to fully capture metabolic health and disease risk (6). Recognizing this limitation, revised obesity classification frameworks have been proposed to enhance diagnostic accuracy and minimize misclassification (7).

As an alternative body composition metric, the fat-to-muscle mass ratio (FMR) has emerged as a tool for evaluating the balance between the adipose tissue and the skeletal muscle (8). Emerging studies have linked FMR to various metabolic disorders, including type 2 diabetes (9), metabolic syndrome (10), coronary artery disease (11), cardiometabolic risks (12), and metabolic dysfunction-associated steatotic liver disease (13), as well as mortality (14). Mechanistically, elevated FMR may contribute to the development of DKD through two interconnected pathways. First, dysfunctional adipose tissue promotes systemic inflammation by releasing pro-inflammatory cytokines such as TNF-*α* and IL-6, which induce glomerular and tubular injury, as well as renal fibrosis. Second, insulin resistance resulting from muscle lipid accumulation and adipokine dysregulation exacerbates glomerular hyperfiltration, endothelial dysfunction, and albuminuria. Together, these processes form a pathogenic cascade, where high FMR induces inflammation and insulin resistance, leading to structural renal damage and driving the progression of DKD. Despite these advancements, the role of FMR in DKD prevalence and mortality remains insufficiently explored.

This study aims to explore the association between FMR and both DKD prevalence and mortality. By elucidating these associations, we seek to provide insights that may inform lifestyle modifications integrating both fat and muscle considerations to mitigate DKD risk and improve patient outcomes.

## Materials and methods

### NHANES analysis

The NHANES is a large-scale, cross-sectional health program conducted under the supervision of the Centers for Disease Control and Prevention in the United States. It systematically gathers extensive data on various aspects of health, nutrition, and lifestyle factors in the general population. By using a scientifically rigorous and standardized methodology, NHANES ensures high-quality data collection. Moreover, the survey is structured around a sophisticated, multistage probability sampling design, enabling the generation of a dataset that accurately reflects the demographic and health characteristics of the US population.

**Figure 1:**
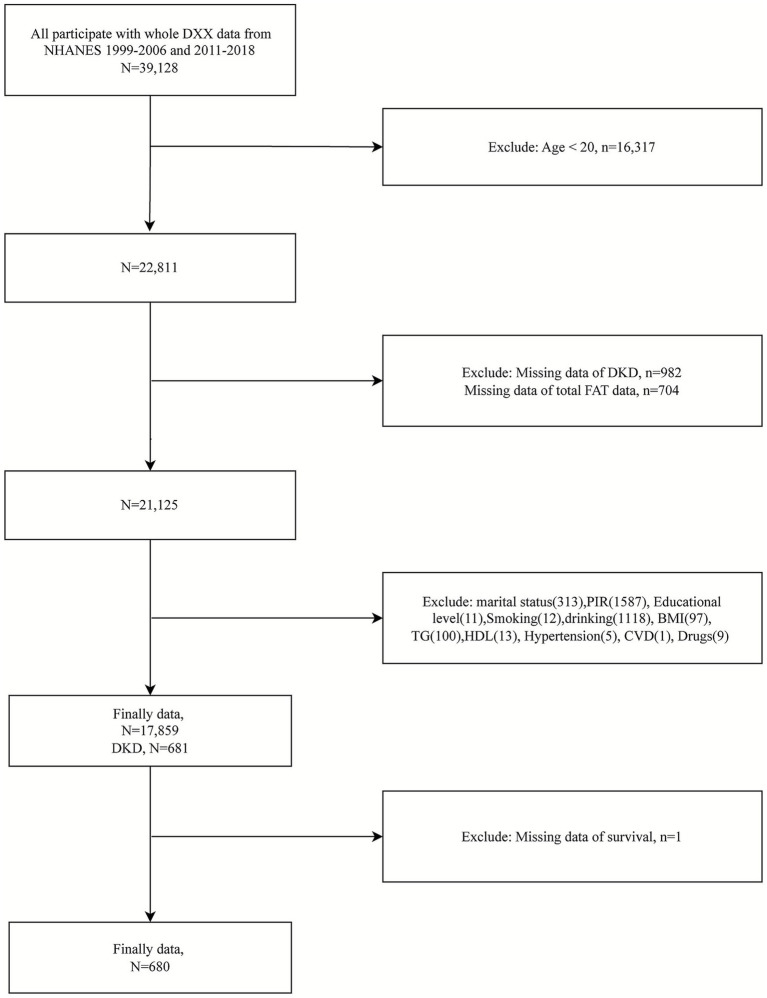
Flowchart of study participants.

For this study, we selected participants with complete body composition data necessary for calculating the FMR. This included individuals with available measurements obtained via dual-energy X-ray absorptiometry (DXA) from NHANES examination data, including body fat percentage and muscle mass. Data were extracted from the 1999–2006 and 2011–2018 NHANES cycles, which provide extensive information on demographics, health status, and body composition. Initially, a total of 39,128 individuals with complete DXA data were identified. After applying the exclusion criteria, 16,317 individuals under 20 years, 982 participants with missing DKD data, 704 participants lacking relevant FMR data, and 3,267 participants missing other essential data were excluded. As a result, 17,859 adults remained in the initial cohort, and finally, 680 individuals diagnosed with DKD fulfilled the criteria and were ultimately included (Figure 1).

### Real-world validation cohort

To validate the findings derived from NHANES, we additionally analyzed an independent hospital-based clinical cohort from the First Affiliated Hospital of Fujian Medical University. This cohort consisted of 94 patients with and without DKD enrolled between March and May 2025. FMR indices, including arm, leg, trunk, and total FMR, were assessed using DXA. Logistic regression models were applied to evaluate the association between FMRs and DKD in this clinical dataset. Model discrimination was assessed using the area under the ROC curve. The real-world validation cohort was approved by the Ethics Committee of First Affiliated Hospital of Fujian Medical University (Approval No.: MTCA, ECFAH of FMU [2015]084–2).

### Variable definition

This study utilized NHANES data with a cross-sectional design for baseline assessments. DKD status was determined cross-sectionally at the time of NHANES survey participation according to the KDIGO 2021 Guidelines, which are based on clinical diagnostic criteria, including the presence of albuminuria or a decrease in eGFR (less than 60 mL/min/1.73 m) in patients with diabetes (15). Albuminuria was identified by a urinary albumin-to-creatinine ratio (UACR) of ≥30 mg/g in individuals with diabetes. The CKD-EPI formula was used to derive eGFR values (16). The FMR was assessed as the ratio of fat mass to muscle mass, with both components assessed using DXA from NHANES physical examination data. Given the clinical significance of regional body composition, FMR was assessed for the total body, trunk, legs, and arms. Participants were categorized into quartiles (Q1–Q4) of FMR, with Q1 (lowest FMR) designated as the reference category. Mortality outcomes were prospectively tracked via linkage to the National Death Index through 31 December 2019, with a median follow-up of 97 months. It is important to note that, while mortality was tracked prospectively, DKD status was assessed cross-sectionally. Therefore, this study design does not constitute an incidence cohort for DKD, and no causal inferences can be drawn between FMR and DKD development. Further definitions and classifications of key variables, including diabetes diagnosis, income-to-poverty ratio (PIR), and causes of death, are provided in Supplementary Table S1. These definitions follow the established NHANES and CDC criteria to ensure consistency across analyses.

### Covariates

The analysis incorporated multiple covariates, including demographic characteristics, lifestyle factors (alcohol consumption, smoking habits, family income-to-poverty ratio, and physical activity), and clinical indicators (BMI, lipid profiles, hypertension status, lipid-lowering medication use, and self-reported CVD).

### Statistical methods

Continuous variables were expressed as mean ± standard error, and categorical variables were expressed as weighted percentages and frequencies. Logistic regression analyses and Cox proportional hazards models were applied to evaluate the association between FMR and DKD onset, as well as its impact on all-cause and CVD mortality. For the analysis of CVD-specific mortality, the competing risk of non-CVD death was accounted for using Fine-Gray hazards models, with results presented as sub-distribution hazard ratios and 95% confidence intervals (CIs). Subgroup analyses were conducted stratified by age (≥60 vs. <60 years), sex, and physical activity (none, moderate, and vigorous). The Kaplan–Meier survival curves were constructed to visualize survival probabilities in DKD patients across FMR groups. For non-linear trends, recursive algorithms were used to detect critical inflection points, and a biphasic Cox framework was applied to assess differential mortality risks. Sensitivity analyses were performed by stratifying participants based on sex, age, physical activity, and DKD status.

To account for the complex survey design of the NHANES dataset, all analyses incorporated the appropriate survey weights, design strata, and primary sampling units, as recommended by the NHANES analytical guidelines. This finding ensures valid standard errors, confidence intervals, and *p*-values for population-level inference.

For the real-world validation cohort, a logistic regression analysis was used to examine the association between FMRs and DKD, with odds ratios (ORs) and 95% CIs reported. Models’ discrimination was evaluated using the area under the ROC curve (AUC). To assess the internal validity and stability of the ROC-derived models, bootstrap resampling with 500 replicates was performed on the NHANES dataset, from which the mean AUC and its 95% confidence interval were calculated.

All analyses were conducted using R version 4.3.1, with a two-sided *p*-value of < 0.05 considered statistically significant.

## Results

### Participant demographics and characteristics

This cohort comprised 680 participants with an average age of 56 years, of whom 57.7% were male. Over a median follow-up period of 97 months, 267 deaths (37.58%) were recorded. Table 1 summarizes the baseline characteristics and laboratory findings, categorized by total-FMR quartiles. Participants in higher FMR quartiles tended to have lower socioeconomic status, engage in less physical activity, exhibit lower triglyceride levels, and experience higher rates of all-cause and CVD mortality than those in lower FMR quartiles (all *p* < 0.05).

**Table 1:** Baseline characteristics according to the FMR quartiles.

Variable	Total	Q1	Q2	Q3	Q4	*p* value
Range		<0.437	(0.437, 0.557)	(0.557, 0.722)	≥0.722	
HbA1c	7.861 ± 0.096	8.217 ± 0.254	7.844 ± 0.177	7.673 ± 0.176	7.726 ± 0.207	0.329
eGFR, ml/min*1.73m^2^	79.782 ± 1.733	84.124 ± 2.613	79.065 ± 2.315	79.617 ± 3.039	76.259 ± 3.496	0.168
uACR, mg/g	326.243 ± 41.047	351.698 ± 68.688	313.834 ± 68.000	266.360 ± 63.983	378.370 ± 113.203	0.754
Age, years	56.838 ± 0.688	54.242 ± 1.090	59.474 ± 1.216	56.587 ± 1.428	57.114 ± 1.285	0.016
PIR	2.546 ± 0.104	2.860 ± 0.168	2.610 ± 0.193	2.405 ± 0.221	2.321 ± 0.144	0.047
BMI, kg/m^2^	31.127 ± 0.378	27.093 ± 0.485	29.795 ± 0.504	32.174 ± 0.904	35.412 ± 0.600	< 0.0001
TG, mmol/L	3.102 ± 0.267	4.469 ± 0.840	3.357 ± 0.550	2.323 ± 0.139	2.323 ± 0.135	0.03
TC, mmol/L	5.288 ± 0.072	5.462 ± 0.152	5.164 ± 0.176	5.128 ± 0.112	5.413 ± 0.112	0.1
HDL, mmol/L	1.216 ± 0.021	1.179 ± 0.041	1.168 ± 0.035	1.204 ± 0.039	1.316 ± 0.034	0.014
Left arm fat, g	1977.505 ± 45.802	1251.068 ± 44.721	1657.036 ± 44.056	2248.408 ± 114.807	2737.859 ± 76.369	< 0.0001
Left arm muscle mass, g	3135.702 ± 62.554	3514.199 ± 90.695	3418.275 ± 104.637	3135.265 ± 161.634	2465.714 ± 54.832	< 0.0001
Right arm fat, g	2027.916 ± 46.016	1312.972 ± 42.585	1703.429 ± 41.310	2289.283 ± 121.941	2791.261 ± 71.138	< 0.0001
Right arm muscle mass, g	3268.732 ± 65.783	3699.444 ± 96.324	3570.327 ± 106.618	3241.942 ± 177.915	2555.832 ± 52.370	< 0.0001
Left leg fat, g	4592.650 ± 91.005	2869.202 ± 65.117	3959.129 ± 103.354	5141.108 ± 222.732	6374.458 ± 215.052	< 0.0001
Left leg muscle mass, g	8352.588 ± 139.562	8695.039 ± 194.165	8688.980 ± 223.893	8487.558 ± 434.445	7513.665 ± 170.661	< 0.0001
Right leg fat, g	4707.727 ± 94.016	2961.276 ± 69.015	4054.824 ± 115.378	5232.889 ± 216.639	6558.574 ± 221.442	< 0.0001
Right leg muscle mass, g	8505.459 ± 137.082	8889.912 ± 200.765	8847.940 ± 220.730	8615.375 ± 429.901	7645.603 ± 170.943	< 0.0001
Trunk fat, g	17047.812 ± 373.096	11605.054 ± 399.606	15759.275 ± 405.524	18912.603 ± 778.694	21809.614 ± 565.376	< 0.0001
Trunk muscle mass, g	28552.367 ± 434.762	28989.060 ± 597.683	29896.215 ± 728.546	29325.494 ± 1215.758	25889.215 ± 508.284	< 0.0001
Total fat, g	31558.059 ± 616.667	21207.867 ± 580.461	28377.773 ± 663.550	35059.280 ± 1393.849	41398.231 ± 1051.045	< 0.0001
Total muscle mass, g	55075.254 ± 850.005	57121.780 ± 1190.443	57809.183 ± 1400.605	56117.563 ± 2464.247	49069.722 ± 950.891	< 0.0001
ARM-FMR	0.672 ± 0.014	0.359 ± 0.007	0.494 ± 0.009	0.736 ± 0.014	1.100 ± 0.018	< 0.0001
LEG-FMR	0.571 ± 0.010	0.336 ± 0.007	0.464 ± 0.009	0.631 ± 0.016	0.851 ± 0.016	< 0.0001
TRUNK-FMR	0.600 ± 0.010	0.393 ± 0.007	0.526 ± 0.005	0.640 ± 0.009	0.840 ± 0.011	< 0.0001
Sex						< 0.0001
Male	378(57.739)	161(94.275)	142(83.240)	69(50.393)	6(3.028)	
Female	302(42.261)	9(5.725)	28(16.760)	101(49.607)	164(96.972)	
Ethnicity						0.684
Mexican American	187(12.345)	49(15.497)	45(11.452)	36(8.976)	57(13.799)	
Non-Hispanic Black	155(14.900)	38(13.703)	33(12.970)	46(16.215)	38(16.591)	
Non-Hispanic White	233(53.447)	50(45.756)	62(53.982)	66(59.365)	55(54.130)	
Other Hispanic	49 (9.555)	14(12.429)	14(10.733)	10(8.326)	11(6.815)	
Other ethnicities	56 (9.753)	19(12.616)	16(10.863)	12(7.118)	9(8.666)	
Marital status						0.123
Not single	404(62.543)	119(68.049)	110(68.332)	92(58.806)	83(55.285)	
Single	276(37.457)	51(31.951)	60(31.668)	78(41.194)	87(44.715)	
Educational level						0.37
<High school	154(14.030)	49(16.254)	37(12.588)	35(16.771)	33(10.146)	
High school	279(44.084)	63(39.354)	64(40.307)	75(47.959)	77(48.371)	
>High school	247(41.886)	58(44.392)	69(47.104)	60(35.271)	60(41.483)	
Smoking						0.079
Never	328(45.496)	67(35.470)	72(43.210)	93(46.841)	96(56.518)	
Former	223(33.127)	57(34.848)	67(35.729)	54(35.960)	45(25.580)	
Now	129(21.377)	46(29.681)	31(21.062)	23(17.199)	29(17.901)	
Alcohol consumption						0.03
Never	128(16.787)	18(11.829)	18(8.233)	44(20.808)	48(25.952)	
Former	193(26.366)	53(24.433)	51(31.410)	42(20.801)	47(29.488)	
Mild	199(31.053)	50(31.539)	53(29.373)	55(36.115)	41(26.583)	
Moderate	51(8.401)	13(9.321)	14(11.539)	11(6.139)	13(6.836)	
Heavy	109(17.393)	36(22.879)	34(19.445)	18(16.136)	21(11.141)	
Physical activity						0.008
No	394(55.073)	88(46.235)	84(45.919)	111(66.773)	111(60.207)	
Moderate	181(26.903)	45(28.748)	54(29.099)	37(20.176)	45(30.340)	
Vigorous	105(18.024)	37(25.017)	32(24.982)	22(13.052)	14(9.453)	
Hypertension						0.121
No	169(26.455)	57(36.606)	42(24.422)	33(23.061)	37(21.944)	
Yes	511(73.545)	113(63.394)	128(75.578)	137(76.939)	133(78.056)	
CVD						0.067
No	515(76.725)	134(82.988)	128(74.124)	130(80.885)	123(68.300)	
Yes	165(23.275)	36(17.012)	42(25.876)	40(19.115)	47(31.700)	
Anti hyperlipidemic						0.636
No	445(62.783)	118(67.236)	107(57.868)	109(64.332)	111(61.453)	
Yes	235(37.217)	52(32.764)	63(42.132)	61(35.668)	59(38.547)	
ACEI/ARB						0.65
No	669(98.452)	168(99.265)	169(98.790)	165(97.383)	167(98.480)	
Yes	11(1.548)	2(0.735)	1(1.210)	5(2.617)	3(1.520)	
ALL-caused death						< 0.0001
No	413(62.416)	150(90.915)	89(51.111)	102(61.928)	72(45.267)	
Yes	267(37.584)	20(9.085)	81(48.889)	68(38.072)	98(54.733)	
CVD-caused death						0.003
No	570(84.233)	160(95.021)	137(78.711)	143(83.339)	130(79.784)	
Yes	110(15.767)	10(4.979)	33(21.289)	27(16.661)	40(20.216)	

FMR, fat-to-muscle mass ratio; HbA1c, glycated hemoglobin; eGFR, estimated glomerular filtration rate; uACR, urinary albumin-to-creatinine ratio; PIR, income-to-poverty ratio; BMI, body mass index; TG, triglycerides; TC, total cholesterol; HDL, high-density lipoprotein; anti-hyperlipidemic: the use of lipid-lowering agents (e.g., statins, fibrates, and ezetimibe); ACEI/ARB, angiotensin-converting enzyme inhibitor/angiotensin II receptor blocker; CVD, cardiovascular disease.

In this study population, the prevalence of DKD was 3.81%. Compared to individuals without DKD, those with DKD had a significantly higher BMI and were older. The proportion of male individuals was also greater in the DKD group than in the non-DKD group. In terms of body composition, patients with DKD exhibited significantly higher arm-FMR, trunk-FMR, and total-FMR levels, while leg-FMR showed no significant differences (Supplementary Table S2).

### Association between FMR and DKD risk

The relationship between FMR and DKD is shown in Supplementary Table S3. A 0.1-unit increase in trunk-FMR corresponded to a 26.6% increase in DKD odds (*OR* = 1.266, 95%*CI*: 1.211–1.322, *p* < 0.0001). The association was still evident with statistical significance (*OR* = 1.347, 95%*CI*: 1.243–1.460, *p* < 0.0001), even after controlling for sex, age, PIR, ethnicity, marital status, and education level. Further adjusting for smoking, alcohol consumption, physical activity, hypertension, CVD, use of lipid-lowering medications, BMI, total cholesterol, triglycerides, and high-density lipoprotein slightly attenuated the association (*OR* = 1.245, 95% *CI*: 1.143–1.353, *p* < 0.0001). Elevated arm-FMR, trunk-FMR, and total-FMR were consistently linked to a higher DKD risk, whereas leg-FMR showed no significant association, even after adjusting for confounders.

### Stratified analysis of FMR and DKD risk across subgroups

**Figure 2:**
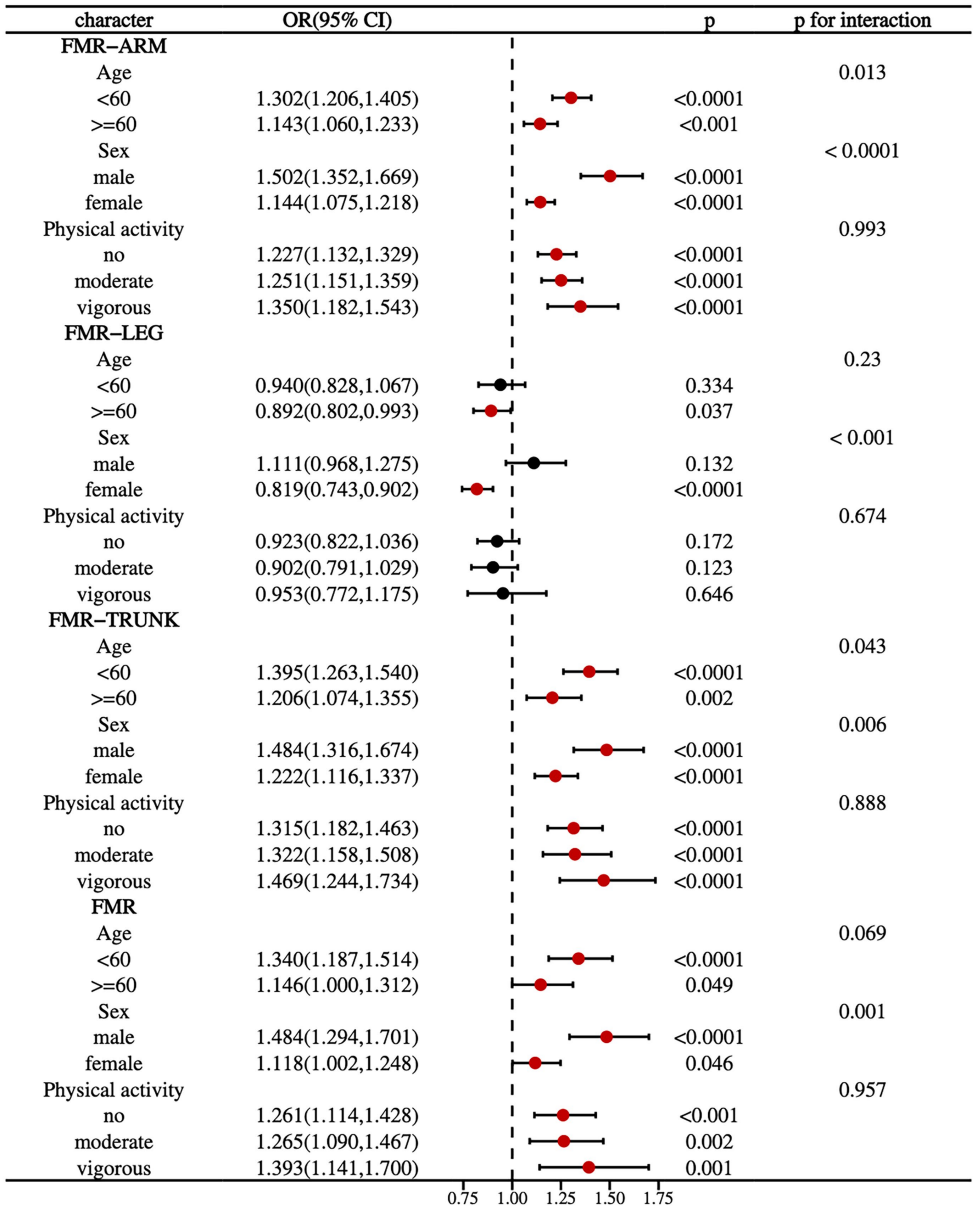
Forest plot for subgroup analysis of DKD prevalence associated with different FMRs.

Stratified subgroup analyses by age, sex, and physical activity demonstrated consistent associations between FMR and DKD prevalence across the majority of subgroups (Figure 2). Significant interactions were observed for the prevalence of DKD in arm-FMR and trunk-FMR when stratified by age and sex. Specifically, younger participants (<60 years) and male individuals with higher FMR exhibited an increased risk of developing DKD (*P* for interaction < 0.05).

### Performance of FMR in identifying DKD

**Figure 3:**
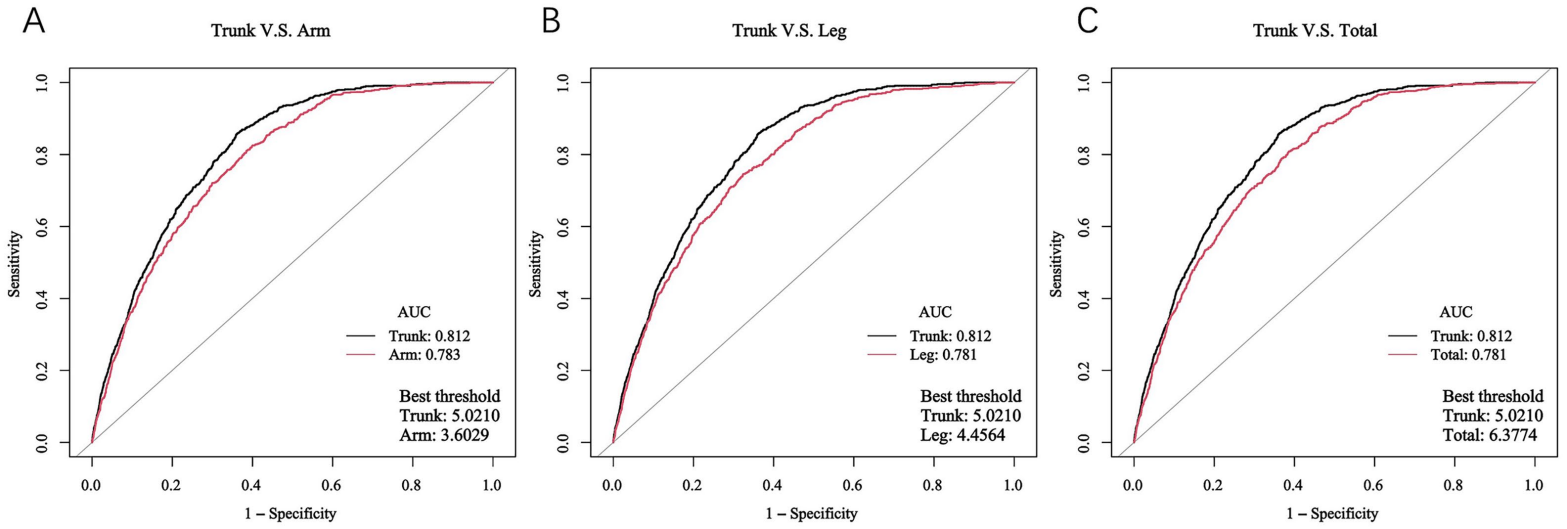
ROC curves of different FMRs in identifying DKD. **(A)** Trunk-FMR: Sensitivity = 85.9%, Specificity = 63.8%, Positive LR = 2.37, Negative LR = 0.22; **(B)** Arm-FMR: Sensitivity = 81.1%, Specificity = 61.5%, Positive LR = 2.11, Negative LR = 0.31; **(C)** Leg-FMR: Sensitivity = 74.6%, Specificity = 67.6%, Positive LR = 2.30, Negative LR = 0.38.

The receiver operating characteristic (ROC) curves were applied to determine the predictive ability of FMR in identifying DKD among T2DM patients, based on FMR combined with significant factors identified in Supplementary Table S4, and are presented in Figure 3. The areas under the ROC curve (AUCs) were 0.812 for trunk-FMR (sensitivity = 85.9%, specificity = 63.8%), 0.783 for arm-FMR (sensitivity = 81.1%, specificity = 61.5%), 0.781 for leg-FMR (sensitivity = 74.6%, specificity = 67.6%), and 0.781 for total-FMR (sensitivity = 80.9%, specificity = 61.3%). Notably, trunk-FMR exhibited the highest AUC, suggesting its superior discriminative ability in identifying DKD risk compared to other FMR indicators.

To internally validate this finding and mitigate concerns about overfitting, we performed bootstrap resampling (500 replicates). This analysis yielded a nearly identical mean AUC of 0.822 (95% CI: 0.810–0.836), confirming the robust discriminative power of trunk-FMR (Supplementary Figure S3).

### Non-linear relationships between different FMRs and the incident DKD

**Figure 4:**
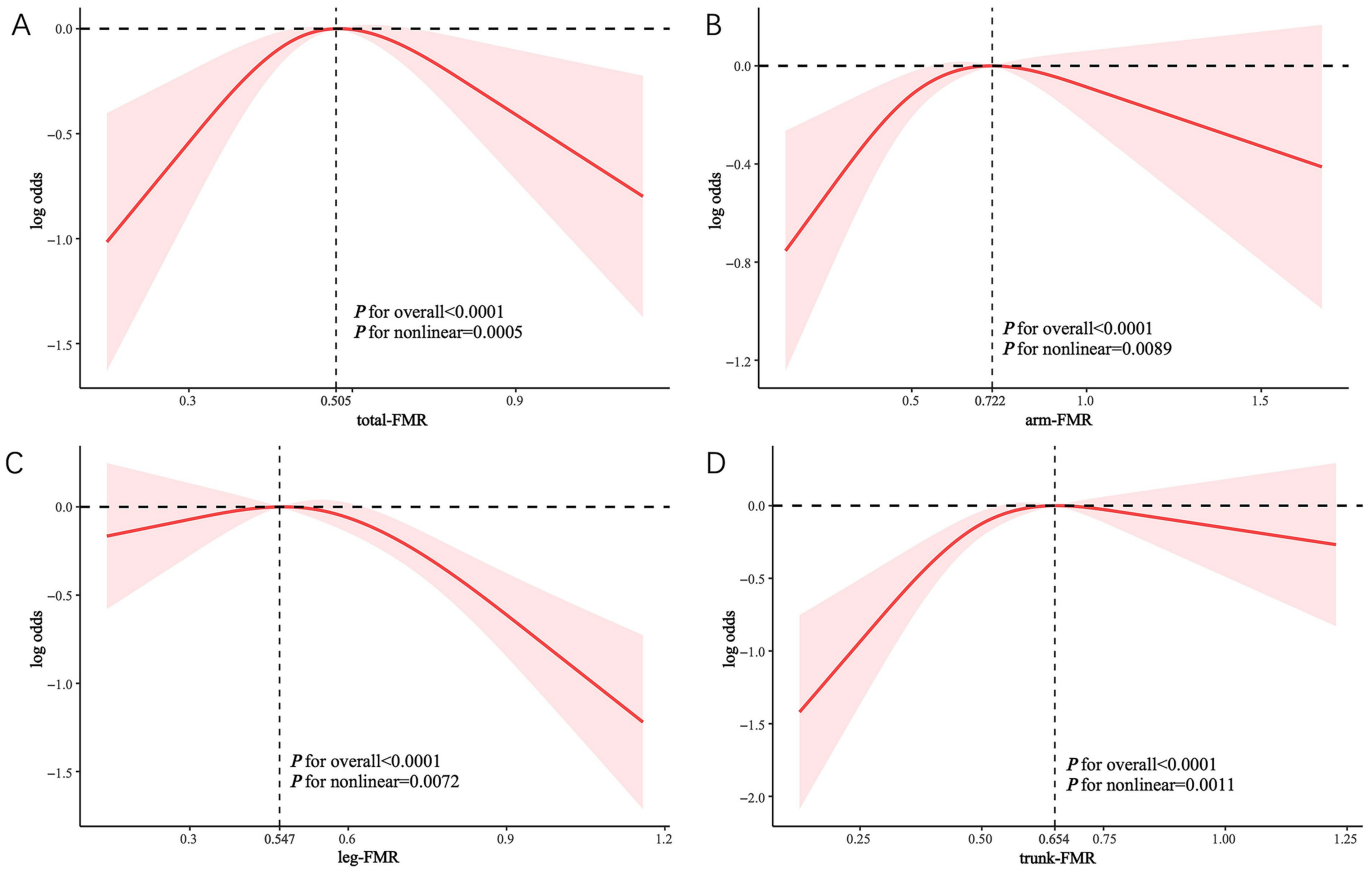
Non-linear associations between different FMRs and the incident DKD, as assessed by RCS analysis. The solid line represents the estimated odds ratio, and the shaded area indicates the 95% confidence interval. The vertical dashed line in each panel indicates the inflection point of the curve, with the exact value as follows: **(A)** total-FMR = 0.505; **(B)** arm-FMR = 0.722; **(C)** leg-FMR = 0.547; **(D)** trunk-FMR = 0.654.

Restricted cubic spline (RCS) analysis revealed non-linear associations between different FMR indices and DKD prevalence. Notably, the adjusted plots demonstrated distinct patterns: an inverted U-shaped relationship between arm-FMR and total-FMR and DKD prevalence (Figures 4A,B), whereas trunk-FMR and leg-FMR exhibited an inverted L-shaped relationship with DKD prevalence (Figures 4C,D).

### The Kaplan–Meier survival curves for all-cause and CVD mortality by FMR quartiles

**Figure 5:**
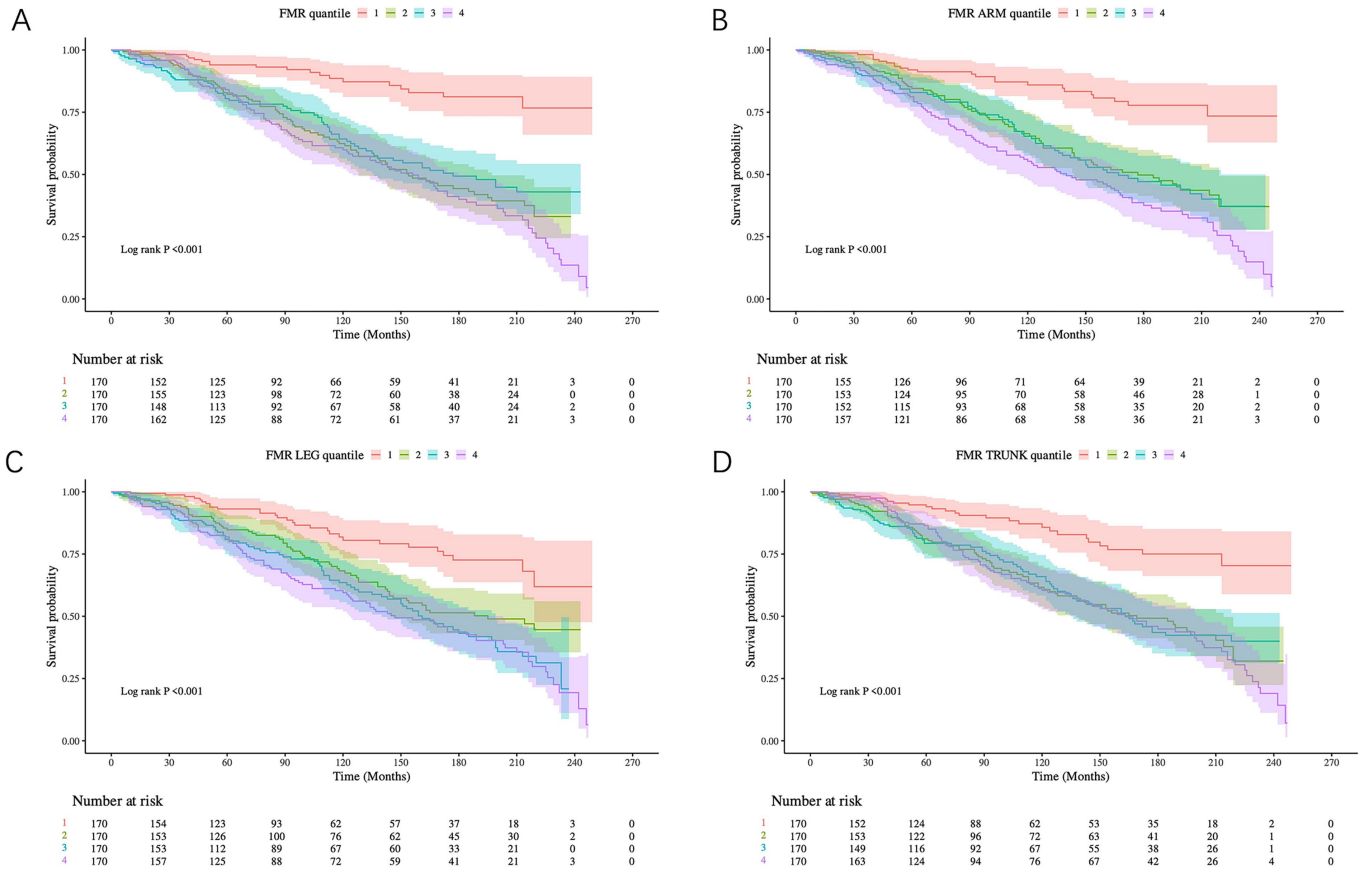
Kaplan–Meier curves displayed for all-cause mortality according to the quartiles of FMR. **(A)** Total-FMR, **(B)** Trunk-FMR, **(C)** Leg-FMR, **(D)** Arm-FMR. The shaded bands around the survival curves represent the 95% confidence intervals. Patient stratification into quartiles was based on the following cut-points derived from the study population: Total-FMR: Q1 (lowest 25%): < 0.42; Q2: 0.42–0.56; Q3: 0.56–0.72; Q4 (highest 25%): ≥ 0.72. Trunk-FMR: Q1: < 0.46; Q2: 0.46–0.58; Q3: 0.58–0.72; Q4: ≥ 0.72. Leg-FMR: Q1: < 0.39; Q2: 0.39–0.53; Q3: 0.53–0.74; Q4: ≥ 0.74. Arm-FMR: Q1: < 0.41; Q2: 0.41–0.59; Q3: 0.59–0.89; Q4: ≥ 0.89.

Over a median follow-up period of 97 months, 267 deaths occurred. K–M survival curves demonstrated that lower FMR quartiles were associated with better survival outcomes for both all-cause and CVD mortality in DKD patients (Figure 5). A similar trend was observed specifically for CVD mortality, as shown in Supplementary Figure S1.

To validate these findings against potential bias from competing risks, we performed a Fine-Gray competing-risk analysis for CVD-specific mortality. The results confirmed a consistent and highly significant association with trunk-FMR, for instance, exhibiting a sub-distribution hazard ratio of 1.269 (95% CI: 1.13–1.42, *p* < 0.0001). The complete results of this analysis are presented in Supplementary Table S7.

### Non-linear associations between different FMRs and all-cause and CVD mortality

**Figure 6:**
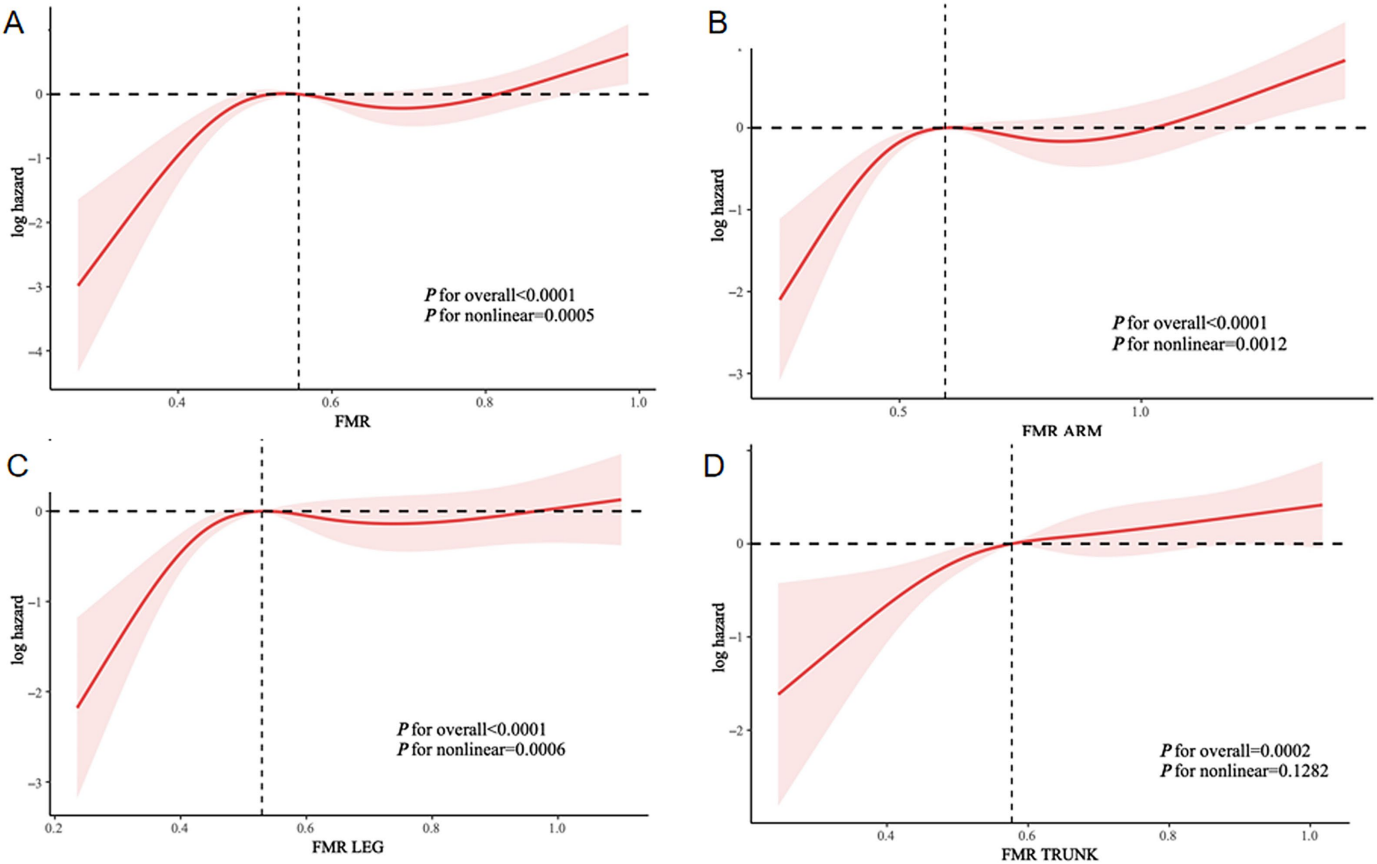
Non-linear associations between different FMRs and all-cause mortality. **(A)** Total-FMR, **(B)** Arm-FMR, **(C)** Leg-FMR, **(D)** Trunk-FMR.

A multivariate analysis identified a non-linear association between FMR and all-cause mortality, as depicted by RCS curves. An inverted L-shaped pattern was evident in the relationship between both total and regional FMR with all-cause mortality (Figure 6), and a comparable trend was observed for CVD mortality (Supplementary Figure S2). These findings indicated non-linear relationships between FMR and mortality risks, emphasizing the complex interplay between body composition and survival outcomes.

### Validation in a real-world cohort

**Figure 7:**
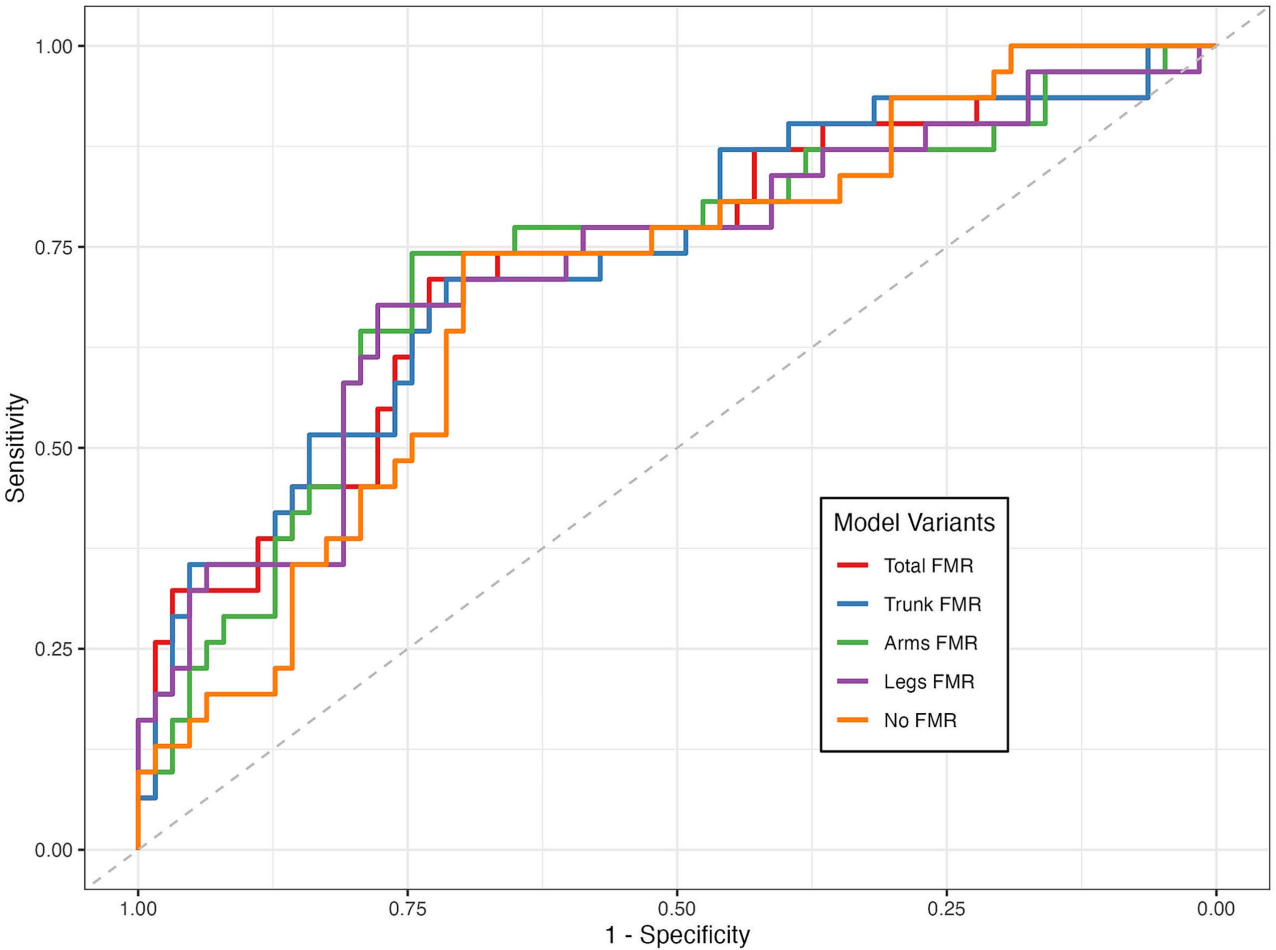
ROC curves for different FMRs in identifying DKD from the real-world cohort.

Based on our previous NHANES study findings, we conducted validation in an independent, real-world, hospital-based cohort, which included 94 patients (Supplementary Table S8). A univariate analysis identified several significant predictive factors for DKD risk: HbA1c (OR = 1.298, 95% CI: 1.053–1.623, *p* = 0.017), total-FMR (OR = 23.132, 95% CI: 1.771–406.856, *p* = 0.022), trunk-FMR (OR = 10.113, 95% CI: 1.404–90.209, *p* = 0.027), and arms-FMR (OR = 7.701, 95% CI: 1.045–63.416, *p* = 0.049 (Supplementary Table S9). Subsequently, we performed a multivariate analysis, incorporating different FMR measurements, HbA1c, and triglycerides (TG) based on the results of the univariate analysis. In the multivariate analysis, trunk-FMR demonstrated the highest discriminatory performance among the site-specific FMR indices (AUC = 0.735, 95% CI: 0.623–0.846), while total-FMR showed the best model fit (AIC = 111.53; Supplementary Table S10; Figure 7). It should be noted that the wide CIs likely reflect the limited sample size and potential variability in clinical measurements. Nevertheless, the direction of the association was consistent with the NHANES analysis, supporting the robustness of trunk-FMR as a correlate of DKD.

## Discussion

In this cohort-based population study, we identified a significant association between both regional and total FMR and the incident DKD. Additionally, DKD patients with lower FMR exhibited better survival outcomes for all-cause and CVD mortality, suggesting that optimizing FMR could be a key strategy for improving DKD prognosis. These findings indicate that FMR, reflecting a relative excess of fat mass relative to muscle mass, could be a critical factor in DKD development. Importantly, the consistency between the nationwide NHANES analysis and the hospital-based validation cohort strengthens the robustness and biological plausibility of trunk FMR as a key predictor of DKD.

Furthermore, our analysis showed that the RCS curves for both total and regional FMR demonstrated a steep increase in mortality risk at higher FMR levels, with a potential inflection point near the upper quartile of the FMR distribution. This pattern was consistent with our K–M survival analysis, which indicated that participants in the highest FMR quartile (Q4) experienced the poorest survival outcomes. Although this inflection point did not represent a definitive diagnostic threshold, the consistent elevation in risk suggests that FMR quartiles, particularly Q4, may serve as a practical tool for clinical risk stratification and for identifying patients who may benefit from more intensive cardiovascular and renal care.

One possible mechanism underlying this relationship is the role of FMR in promoting inflammation. Excessive fat accumulation, commonly associated with high FMR, can contribute to persistent low-grade inflammation (17). Under obesity-related conditions, adipose tissue secretes a range of pro-inflammatory cytokines (18). These cytokines could stimulate inflammatory cascades within the kidneys, facilitating macrophage and T lymphocyte infiltration into renal tissues (19). This inflammatory response can induce glomerular and tubular damage, promote extracellular matrix production, and ultimately drive the onset and progression of DKD (20). The pro-inflammatory and metabolic derangements associated with a high FMR likely converge on the proximal tubule, directly aggravating the pathways of oxidative stress and interstitial fibrosis that are central to the pathogenesis of diabetic tubulopathy (21). Furthermore, this pro-inflammatory and metabolic dysregulation induced by high FMR may foster a cellular environment susceptible to ferroptosis, an iron-dependent regulated cell death driven by lipid peroxidation that is increasingly linked to diabetic kidney damage pathogenesis (22). Additionally, the pro-inflammatory state driven by high FMR might be further amplified by concurrent reductions in short-chain fatty acids (SCFAs), as a diminished SCFA pool fails to activate GPR-mediated anti-inflammatory pathways, thereby creating a permissive environment for renal inflammation and fibrosis (23). Our previous findings demonstrated that systemic inflammation, as assessed by the systemic inflammatory response index, synergistically amplifies mortality risks in individuals with advanced cardiovascular–kidney–metabolic stages, underscoring the pivotal role of inflammation in chronic disease progression (24). This finding suggests that the inflammatory pathways associated with high FMR may similarly contribute to the pathogenesis of DKD.

Another possible mechanism is insulin resistance, which is frequently associated with high FMR (25). Interestingly, our finding that a high fat-to-muscle ratio promotes DKD risk aligns with emerging evidence on oxidative stress, where a higher oxidative balance score (reflecting a preponderance of antioxidants) is protective. This finding suggests that the pro-inflammatory and insulin-resistant state driven by adverse body composition may be mechanistically linked to oxidative stress pathways in the pathogenesis of DKD (26). Insulin resistance in muscle and adipose tissue impairs glucose uptake and metabolism, leading to compensatory hyperinsulinemia. In turn, hyperinsulinemia stimulates the renin-angiotensin-aldosterone pathway, resulting in increased glomerular filtration and elevated blood pressure (27). Prolonged glomerular hyperfiltration imposes hemodynamic strain on glomerular capillaries, disrupts the glomerular filtration barrier, and promotes albuminuria, an early indicator of diabetic nephropathy (28). Additionally, insulin resistance impairs kidney function by altering glucose reabsorption and metabolism, further contributing to renal damage and DKD progression (29). High-calorie diets and sedentary lifestyles that promote a high FMR create a systemic milieu that concurrently exacerbates oxidative stress, impairs renal-protective SCFA signaling, and dysregulates pro-fibrotic pathways such as TGF-*β*/Smad, thereby accelerating the progression of diabetic kidney disease through multiple interconnected mechanisms (30).

Previous studies on body composition and DKD mainly focused on BMI or individual fat and muscle mass (31, 32). By utilizing FMR as an integrated indicator of body composition, our study provides a more comprehensive perspective. Consistent with previous research linking obesity to DKD (33, 34), our findings indicate that elevated FMR is linked to a higher likelihood of developing DKD. Moreover, several studies have directly examined the role of FMR in DKD, and our findings further expand the current understanding of this relationship. Notably, the strong association between trunk-FMR and DKD observed in this study underscores the clinical significance of regional fat distribution, emphasizing the need for a more nuanced assessment of body composition in DKD risk stratification. Trunk-FMR primarily reflects central adiposity, which is closely associated with visceral fat accumulation. Visceral fat-derived factors directly promote systemic inflammation and insulin resistance, both of which are key pathways in the pathogenesis of DKD. Compared to peripheral fat, visceral fat exhibits greater metabolic activity and is more strongly associated with dysregulated lipid metabolism, further contributing to endothelial injury and renal tubular damage. These mechanisms collectively explain why trunk-FMR, as a marker of central adiposity and visceral fat burden, showed superior predictive power for DKD risk and mortality in our study. In addition, the robustness and generalizability of these findings were further supported by an independent hospital-based cohort, which provided real-world validation consistent with the NHANES results.

While this study provides valuable insights, certain limitations should be acknowledged. First, the data were derived from the NHANES database, which primarily represented the US population. Consequently, these findings may have limited generalizability to populations with diverse genetic backgrounds, dietary patterns, and lifestyle factors. Second, the cross-sectional design precludes causal inferences between FMR and DKD. Third, since only baseline body composition was assessed, the impact of long-term fluctuations in FMR on DKD progression remains unclear. Finally, despite extensive adjustments for confounders, the possibility of residual confounding cannot be completely excluded. In addition, the real-world validation cohort focused on DKD prevalence and did not include mortality follow-up, so it does not verify the associations between FMR and all-cause/CVD mortality observed in the NHANES analysis. Future long-term follow-up of this clinical cohort is planned to assess mortality outcomes and further validate the prognostic value of FMR. Although the hospital-based validation cohort strengthened our findings, its relatively small sample size and the lack of long-term mortality follow-up may have contributed to limited statistical power and should be considered when interpreting the generalizability of its findings.

To address the current limitations and confirm the potential causal role of FMR in DKD, future research should prioritize longitudinal cohort studies to better establish the temporal relationship between FMR and DKD onset and progression. Additionally, intervention trials aimed at modifying body composition, such as through structured exercise or nutritional programs, would help clarify whether improving FMR can causally reduce DKD risk and improve clinical outcomes. Moreover, mechanistic studies exploring the biological pathways underlying the FMR-DKD association could provide a theoretical basis for targeted interventions.

Clinically, trunk-FMR could serve as a complementary marker to BMI for DKD risk stratification. While BMI reflects overall adiposity, trunk-FMR specifically captures central fat distribution, which is more strongly associated with visceral fat-mediated inflammation and insulin resistance. Clinicians could combine elevated BMI (≥25 kg/m^2^) and high trunk-FMR (≥0.6) to identify patients who warrant more intensive screening, such as annual UACR and eGFR assessments. By integrating trunk-FMR with BMI, a more nuanced understanding of body composition-related DKD risk is achieved, enabling targeted, mechanism-based prevention strategies. Early interventions aimed at improving body composition, including maintaining a well-balanced diet and engaging in consistent physical activity, may help optimize FMR and potentially contribute to DKD prevention. Additionally, comprehensive management strategies, incorporating strict blood pressure regulation, glycemic control, and lipid-lowering therapy, may further reduce DKD risk. Future research should focus on longitudinal studies to determine the causal link between FMR and DKD. Moreover, multicenter investigations with expanded sample sizes are necessary to validate our findings and investigate the fundamental mechanisms driving this association.

Overall, this NHANES-based analysis identified a significant association between FMR and DKD prevalence, as well as all-cause and CVD mortality. Importantly, these associations were validated in an independent hospital-based cohort, supporting the robustness and generalizability of our findings. These results offer key insights into how body composition affects DKD progression, emphasizing the importance of maintaining a balanced fat-to-muscle ratio for DKD prevention. To strengthen these findings, additional research is required to confirm the associations observed and to explore optimized preventive and therapeutic strategies for DKD management.

## Data Availability

The raw data supporting the conclusions of this article will be made available by the authors, without undue reservation.
