# Supplementary material for: Association between fat-to-muscle ratio and diabetic kidney disease: a nationwide NHANES analysis with real-world validation
Source: Front Nutr. 2025 Nov 28;12:1700718. doi: 10.3389/fnut.2025.1700718 (PMC12699349; doi:10.3389/fnut.2025.1700718)
Supplement: Supplementary file 1 [file Table_1.docx]

**Table S1**. Key Definitions and Classifications of Study Variables

|  | **Definition** |
| --- | --- |
| Diabetes mellitus | Diabetes mellitus was diagnosed following the American Diabetes Association guidelines, which include fasting blood glucose (FBG) levels ≥7.0 mmol/L, glycosylated hemoglobin A1c (HbA1c) levels ≥6.5%, random blood glucose ≥11.1 mmol/L, two-hour OGTT blood glucose ≥11.1 mmol/L, or current use of diabetes medications or insulin. |
| Causes of death | Causes of death were classified based on the International Classification of Diseases, Tenth Revision (ICD-10), where all-cause mortality encompassed deaths from any underlying cause, and CVD mortality included deaths identified by ICD-10 codes I00-I09, I11, I13, I20-I51, and I60-I69. |
| Income-to-poverty ratio (PIR) | PIR was categorized as low (<3.0) or high (≥3.0) income. Physical activity levels were classified as none, moderate, or vigorous. |

**TableS2**.Participant demographics and characteristics.

|  | **Total** | **No-DKD** | **DKD** | **Pvalue** |
| --- | --- | --- | --- | --- |
| **n** | **17859** | **17178** | **681** |  |
| **HbA1c** | 5.300(5.100,5.600) | 5.300(5.100,5.500) | 7.200(6.200,9.100) | < 0.0001 |
| **eGFR, ml/min*1.73m^2^** | 99.237(85.375,111.461) | 99.390(85.869,111.741) | 83.816(55.047,101.142) | < 0.0001 |
| **uACR, mg/g** | 5.830(3.986,10.068) | 5.714(3.951, 9.619) | 60.261(33.496,175.254) | < 0.0001 |
| **Age, years** | 41.000(30.000,52.000) | 41.000(30.000,51.000) | 55.000(47.000,67.000) | < 0.0001 |
| **PIR** | 3.140(1.570,5.000) | 3.160(1.580,5.000) | 2.160(1.190,4.000) | < 0.0001 |
| **BMI, kg/m^2^** | 26.900(23.600,30.800) | 26.800(23.520,30.670) | 30.160(26.890,35.110) | < 0.0001 |
| **TG, mmol/L** | 1.276(0.858,1.976) | 1.264(0.847,1.942) | 2.123(1.321,3.093) | < 0.0001 |
| **TC, mmol/L** | 4.991(4.370,5.715) | 4.991(4.370,5.715) | 5.090(4.396,6.025) | 0.008 |
| **HDL, mmol/L** | 1.290(1.060,1.580) | 1.290(1.070,1.600) | 1.140(0.980,1.400) | < 0.0001 |
| **left arm fat, g** | 1431.200(1088.200,1885.800) | 1423.500(1084.600,1876.700) | 1799.900(1366.800,2383.700) | < 0.0001 |
| **left arm muscle mass, g** | 2884.400(2077.300,3757.900) | 2879.700(2072.300,3757.400) | 3007.600(2366.400,3816.200) | 0.008 |
| **right arm fat, g** | 4240.500(3276.000,5473.100) | 4243.300(3275.300,5472.800) | 4143.700(3280.800,5492.900) | < 0.0001 |
| **right arm muscle mass, g** | 7993.000(6470.400,9534.200) | 7991.300(6469.500,9527.800) | 8133.800(6605.600,9886.400) | 0.036 |
| **left leg fat, g** | 1475.600(1120.900,1928.000) | 1468.600(1113.700,1913.800) | 1857.300(1445.200,2416.700) | 0.471 |
| **left leg muscle mass, g** | 3054.000(2206.100,3949.500) | 3052.400(2200.500,3948.600) | 3128.700(2433.500,4016.000) | 0.066 |
| **right leg fat, g** | 4350.100(3369.800,5629.100) | 4353.700(3372.000,5629.500) | 4243.200(3264.600,5604.300) | 0.542 |
| **right leg muscle mass, g** | 8134.300(6592.600,9720.100) | 8130.000(6589.800,9711.100) | 8346.400(6705.700,9986.400) | 0.074 |
| **trunk fat, g** | 12209.300(8624.700,16210.900) | 12113.700(8588.300,16060.400) | 16160.800(12573.100,20944.300) | < 0.0001 |
| **trunk muscle mass, g** | 25301.900(21050.700,29741.300) | 25242.200(21018.600,29658.200) | 27812.100(23269.800,33254.600) | < 0.0001 |
| **total fat, g** | 24963.900(19297.100,31937.700) | 24816.400(19211.900,31770.500) | 29828.700(23633.200,38007.500) | < 0.0001 |
| **total muscle mass, g** | 50791.500(41462.000,59978.900) | 50724.900(41414.400,59882.000) | 53994.600(44217.900,64604.900) | < 0.0001 |
| **ARM-FMR** | 0.490(0.338,0.781) | 0.487(0.336,0.778) | 0.599(0.418,0.891) | < 0.0001 |
| **LEG-FMR** | 0.541(0.384,0.786) | 0.542(0.383,0.788) | 0.529(0.387,0.722) | 0.059 |
| **TRUNK-FMR** | 0.478(0.357,0.632) | 0.475(0.355,0.630) | 0.574(0.466,0.723) | < 0.0001 |
| **TOTAL-FMR** | 0.494(0.378,0.671) | 0.492(0.376,0.670) | 0.562(0.439,0.712) | < 0.0001 |
| **Sex** |  |  |  | 0.039 |
| male | 9464(52.128) | 9086(51.990) | 378(57.687) |  |
| female | 8395(47.872) | 8092(48.010) | 303(42.313) |  |
| **Race** |  |  |  | < 0.0001 |
| Mexican American | 3475(8.769) | 3288( 8.681) | 187(12.334) |  |
| Non-Hispanic Black | 3325( 9.555) | 3169( 9.421) | 156(14.977) |  |
| Non-Hispanic White | 8003(69.029) | 7770(69.417) | 233(53.398) |  |
| Other Hispanic | 1213( 5.991) | 1164(5.902) | 49(9.547) |  |
| Other Race | 1843( 6.656) | 1787(6.579) | 56(9.744) |  |
| **Marital status** |  |  |  | 0.393 |
| no-single | 11128(64.853) | 10723(64.909) | 405(62.577) |  |
| single | 6731(35.147) | 6455(35.091) | 276(37.423) |  |
| **Educational level** |  |  |  | < 0.0001 |
| <high school | 1750( 4.616) | 1596( 4.383) | 154(14.017) |  |
| high school | 6461(33.744) | 6182(33.488) | 279(44.044) |  |
| >high school | 9648(61.640) | 9400(62.129) | 248(41.939) |  |
| **Smoking** |  |  |  | < 0.0001 |
| never | 9786(53.810) | 9458(54.017) | 328(45.455) |  |
| former | 3892(22.259) | 3668(21.988) | 224(33.187) |  |
| now | 4181(23.931) | 4052(23.995) | 129(21.358) |  |
| **Drinking** |  |  |  | < 0.0001 |
| never | 2270(10.140) | 2142( 9.976) | 128(16.772) |  |
| former | 2554(12.013) | 2361(11.658) | 193(26.342) |  |
| mild | 5924(34.773) | 5724(34.864) | 200(31.115) |  |
| moderate | 2931(18.502) | 2880(18.753) | 51( 8.393) |  |
| heavy | 4180(24.571) | 4071(24.749) | 109(17.378) |  |
| **Physical activity** |  |  |  | < 0.0001 |
| no | 8040(39.822) | 7645(39.448) | 395(55.114) |  |
| moderate | 4534(27.017) | 4353(27.024) | 181(26.879) |  |
| vigorous | 5283(33.147) | 5178(33.527) | 105(18.008) |  |
| **Hypertension** |  |  |  | < 0.0001 |
| No | 12040(71.003) | 11871(72.108) | 169(26.431) |  |
| Yes | 5819(28.997) | 5307(27.892) | 512(73.569) |  |
| **CVD** |  |  |  | < 0.0001 |
| No | 16727(95.224) | 16211(95.682) | 516(76.746) |  |
| Yes | 1132( 4.776) | 967( 4.318) | 165(23.254) |  |
| **anti Hyperlipidemic** |  |  |  | < 0.0001 |
| No | 16099(90.768) | 15653(91.461) | 446(62.816) |  |
| Yes | 1760( 9.232) | 1525( 8.539) | 235(37.184) |  |
| **ACEI/ARB** |  |  |  | 0.821 |
| No | 17625(98.569) | 16955(98.571) | 670(98.454) |  |
| Yes | 234( 1.431) | 223(1.429) | 11(1.546) |  |

**Table S3.** Logistic analysis between different FMRs and the DKD prevalence.

|  | **Crude Model**  **OR (95%CI)** | **P-value** | **Model 1**  **OR (95%CI)** | **P-value** | **Model 2**  **OR (95%CI)** | **P-value** |
| --- | --- | --- | --- | --- | --- | --- |
| **ARM FMR, every 0.1 point** | 1.111 (1.082, 1.141) | <0.0001 | 1.258 (1.185, 1.336) | <0.0001 | 1.188 (1.117, 1.264) | <0.0001 |
| **ARM quartile** |  |  |  |  |  |  |
| **Q1** | Ref. |  | Ref. |  | Ref. |  |
| **Q2** | 2.729 (1.791, 4.158) | <0.0001 | 2.278 (1.454, 3.570) | 0.0005 | 2.015 (1.304, 3.115) | 0.0021 |
| **Q3** | 2.784 (1.844, 4.204) | <0.0001 | 4.511 (2.652, 7.670) | <0.0001 | 3.417 (1.972, 5.921) | <0.0001 |
| **Q4** | 3.343 (2.311, 4.835) | <0.0001 | 7.949 (4.064, 15.549) | <0.0001 | 5.059 (2.545, 10.056) | <0.0001 |
| **P for trend** | <0.0001 |  | <0.0001 |  | <0.0001 |  |
| **LEG FMR, every 0.1 point** | 0.967 (0.934, 1.002) | 0.0638 | 0.937 (0.859, 1.021) | 0.1418 | 0.905 (0.832, 0.984) | 0.0221 |
| **LEG quartile** |  |  |  |  |  |  |
| **Q1** | Ref. |  | Ref. |  | Ref. |  |
| **Q2** | 1.248 (0.941, 1.655) | 0.1264 | 1.122 (0.823, 1.530) | 0.4681 | 0.987 (0.718, 1.358) | 0.9375 |
| **Q3** | 1.273 (0.977, 1.660) | 0.0769 | 1.351 (0.845, 2.159) | 0.2118 | 1.148 (0.727, 1.812) | 0.5543 |
| **Q4** | 0.793 (0.605, 1.038) | 0.0943 | 0.709 (0.414, 1.213) | 0.2125 | 0.575 (0.338, 0.979) | 0.0444 |
| **P for trend** | 0.1184 |  | 0.4874 |  | 0.1465 |  |
| **TRUNK FMR, every 0.1 point** | 1.266 (1.211, 1.322) | <0.0001 | 1.347 (1.243, 1.460) | <0.0001 | 1.245 (1.145, 1.355) | <0.0001 |
| **TRUNK quartile** |  |  |  |  |  |  |
| **Q1** | Ref. |  | Ref. |  | Ref. |  |
| **Q2** | 2.875 (1.919, 4.307) | <0.0001 | 2.222 (1.442, 3.423) | 0.0005 | 1.976 (1.283, 3.043) | 0.0026 |
| **Q3** | 4.575 (2.990, 7.001) | <0.0001 | 3.545 (2.170, 5.791) | <0.0001 | 2.702 (1.636, 4.462) | 0.0002 |
| **Q4** | 5.191 (3.470, 7.764) | <0.0001 | 5.136 (3.015, 8.748) | <0.0001 | 3.416 (1.963, 5.942) | <0.0001 |
| **P for trend** | <0.0001 |  | <0.0001 |  | 0001 |  |
| **TOTAL FMR, every 0.1 point** | 1.153 (1.105, 1.204) | <0.0001 | 1.298 (1.179, 1.429) | <0.0001 | 1.185 (1.075, 1.307) | 0.0009 |
| **TOTAL quartile** |  |  |  |  |  |  |
| **Q1** | Ref. |  | Ref. |  | Ref. |  |
| **Q2** | 2.428 (1.716, 3.434) | <0.0001 | 2.121 (1.473, 3.054) | 0.0001 | 1.862 (1.301, 2.667) | 0.0010 |
| **Q3** | 2.913 (2.059, 4.120) | <0.0001 | 3.430 (2.185, 5.384) | <0.0001 | 2.617 (1.632, 4.198) | 0.0001 |
| **Q4** | 2.885 (2.070, 4.021) | <0.0001 | 4.200 (2.502, 7.052) | <0.0001 | 2.680 (1.587, 4.524) | 0.0004 |
| **P for trend** | <0.0001 |  | <0.0001 |  | 0.0004 |  |

Crude Model: No-adjust

Model 1: Age, Sex, Race, PIR, marital status, Educational Level

Model 2: Model 1, Smoke, Drinking, Physical Activity, Hypertension, CVD, Antihyperlipidemic Drugs, BMI, TC, TG and HDL, ACEI/ARB

**Table S4.** Univariate analysis of the risk factors for the DKD prevalence.

|  | OR (95%CI) |
| --- | --- |
| **Age, year** | 1.070 (1.063, 1.077) <0.0001 |
| **Sex** |  |
| male | Ref. |
| female | 0.794 (0.640, 0.986) 0.0392 |
| **Race** |  |
| Mexican American | Ref. |
| Non-Hispanic Black | 1.119 (0.858, 1.460) 0.4094 |
| Non-Hispanic White | 0.541 (0.402, 0.730) 0.0001 |
| Other Hispanic | 1.138 (0.714, 1.815) 0.5872 |
| Other Race | 1.042 (0.658, 1.652) 0.8600 |
| **Marital status** |  |
| No-single | Ref. |
| Single | 1.106 (0.878, 1.393) 0.3927 |
| **PIR** | 0.820 (0.762, 0.882) <0.0001 |
| **Educational level** |  |
| <high school | Ref. |
| High School | 0.411 (0.305, 0.554) <0.0001 |
| >High school | 0.211 (0.158, 0.282) <0.0001 |
| **Smoking** |  |
| never | Ref. |
| former | 1.794 (1.407, 2.287) <0.0001 |
| now | 1.058 (0.823, 1.359) 0.6611 |
| **Drinking** |  |
| never | Ref. |
| former | 1.344 (0.983, 1.838) 0.0666 |
| mild | 0.531 (0.388, 0.726) 0.0001 |
| moderate | 0.266 (0.183, 0.388) <0.0001 |
| heavy | 0.418 (0.280, 0.623) <0.0001 |
| **Physical activity** |  |
| no | Ref. |
| moderate | 0.712 (0.564, 0.899) 0.0051 |
| vigorous | 0.384 (0.293, 0.504) <0.0001 |
| **HbA1c** | 2.520(2.332,2.724) <0.0001 |
| **TG** | 1.163 (1.081, 1.252) 0.0001 |
| **TC** | 1.166 (1.056, 1.287) 0.0029 |
| **HDL** | 0.330 (0.223, 0.488) <0.0001 |
| **BMI** | 1.091 (1.076, 1.107) <0.0001 |
| **Hypertension** |  |
| No | Ref. |
| Yes | 7.196 (5.804, 8.921) <0.0001 |
| **CVD** |  |
| No | Ref. |
| Yes | 6.715 (5.105, 8.831) <0.0001 |
| **Antihyperlipidemic Drugs** |  |
| No | Ref. |
| Yes | 6.340 (5.073, 7.924) <0.0001 |
| **ACEI/ARB** |  |
| No | Ref. |
| Yes | 1.084 (0.540, 2.175) 0.8215 |

**Table S5** Cox regression analysis between different FMRs and all-caused mortality.

| **ALL** | **Crude Model**  **HR (95%CI)** | **P-value** | **Model 1**  **HR (95%CI)** | **P-value** | **Model 2**  **HR (95%CI)** | **P-value** |
| --- | --- | --- | --- | --- | --- | --- |
| **ARM FMR, every 0.1 point** | 1.130(1.085,1.177) | <0.0001 | 1.270(1.178,1.369) | <0.0001 | 1.274(1.180,1.375) | <0.0001 |
| **ARM quartile** |  |  |  |  |  |  |
| **Q1** | ref | ref | ref | ref | ref | ref |
| **Q2** | 3.551(1.942,6.494) | <0.0001 | 2.421(1.326, 4.420) | 0.004 | 2.640(1.371, 5.085) | 0.004 |
| **Q3** | 4.608(2.582,8.222) | <0.0001 | 4.926(2.623, 9.250) | <0.0001 | 5.791(3.097,10.827) | <0.0001 |
| **Q4** | 5.404(3.305,8.835) | <0.0001 | 10.546(5.484,20.279) | <0.0001 | 12.078(6.109,23.878) | <0.0001 |
| **P for trend** |  | <0.0001 |  | <0.0001 |  | <0.0001 |
| **LEG FMR, every 0.1 point** | 1.158(1.094,1.226) | <0.0001 | 1.195(1.079,1.324) | <0.001 | 1.203(1.081,1.338) | <0.001 |
| **LEG quartile** |  |  |  |  |  |  |
| **Q1** | ref | ref | ref | ref | ref | ref |
| **Q2** | 2.737(1.624,4.612) | <0.001 | 2.315(1.428,3.752) | <0.001 | 2.526(1.508,4.231) | <0.001 |
| **Q3** | 3.614(2.241,5.830) | <0.0001 | 3.314(2.155,5.095) | <0.0001 | 3.712(2.382,5.786) | <0.0001 |
| **Q4** | 3.654(2.285,5.844) | <0.0001 | 3.085(1.743,5.460) | <0.001 | 3.376(1.883,6.052) | <0.0001 |
| **P for trend** |  | <0.0001 |  | <0.001 |  | <0.0001 |
| **TRUNK FMR, every 0.1 point** | 1.175(1.109,1.245) | <0.0001 | 1.221(1.113,1.340) | <0.0001 | 1.230(1.118,1.353) | <0.0001 |
| **TRUNK quartile** |  |  |  |  |  |  |
| **Q1** | ref | ref | ref | ref | ref | ref |
| **Q2** | 2.521(1.308,4.859) | 0.006 | 2.350(1.359,4.064) | 0.002 | 2.851(1.627,4.995) | <0.001 |
| **Q3** | 2.596(1.377,4.892) | 0.003 | 2.326(1.281,4.223) | 0.006 | 2.651(1.441,4.877) | 0.002 |
| **Q4** | 3.205(1.822,5.637) | <0.0001 | 3.126(1.671,5.849) | <0.001 | 3.570(1.880,6.780) | <0.001 |
| **P for trend** |  | <0.0001 |  | <0.001 |  | <0.001 |
| **TOTAL FMR, every 0.1 point** | 1.215(1.139,1.295) | <0.0001 | 1.358(1.201,1.534) | <0.0001 | 1.371(1.212,1.550) | <0.0001 |
| **TOTAL quartile** |  |  |  |  |  |  |
| **Q1** | ref | ref | ref | ref | ref | ref |
| **Q2** | 5.618(3.200, 9.862) | <0.0001 | 3.887(2.329, 6.486) | <0.0001 | 4.242(2.496, 7.210) | <0.0001 |
| **Q3** | 4.408(2.181, 8.910) | <0.0001 | 4.229(2.126, 8.411) | <0.0001 | 5.001(2.511, 9.958) | <0.0001 |
| **Q4** | 6.391(3.624,11.270) | <0.0001 | 8.133(3.688,17.934) | <0.0001 | 8.896(3.978,19.892) | <0.0001 |
| **P for trend** |  | <0.0001 |  | <0.0001 |  | <0.0001 |

Crude Model: No-adjust

Model 1: Age, Sex, Race, PIR, marital status, Educational Level

Model 2: Model 1, Smoke, Drinking, Physical Activity, Hypertension, CVD, Antihyperlipidemic Drugs, BMI, TC, TG and HDL

**Table S6** Cox regression analysis between different FMRs and CVD mortality.

|  | **Crude Model**  **HR (95%CI)** | **P-value** | **Model 1**  **HR (95%CI)** | **P-value** | **Model 2**  **HR (95%CI)** | **P-value** |
| --- | --- | --- | --- | --- | --- | --- |
| **ARM FMR, every 0.1 point** | 1.115(1.047,1.187) | <0.001 | 1.313(1.174,1.467) | <0.0001 | 1.321(1.166,1.497) | <0.0001 |
| **ARM quartile** |  |  |  |  |  |  |
| **Q1** | ref | ref | ref | ref | ref | ref |
| **Q2** | 3.245(1.493, 7.052) | 0.003 | 2.242(1.011, 4.968) | 0.047 | 2.323(1.076, 5.017) | 0.032 |
| **Q3** | 4.616(2.001,10.649) | <0.001 | 6.024(2.227,16.294) | <0.001 | 6.292(2.408,16.443) | <0.001 |
| **Q4** | 4.513(2.146, 9.490) | <0.0001 | 12.757(4.760,34.185) | <0.0001 | 13.028(4.877,34.801) | <0.0001 |
| **P for trend** |  | <0.001 |  | <0.0001 |  | <0.0001 |
| **LEG FMR, every 0.1 point** | 1.138(1.037,1.248) | 0.006 | 1.244(1.063,1.456) | 0.007 | 1.236(1.046,1.461) | 0.013 |
| **LEG quartile** |  |  |  |  |  |  |
| **Q1** | ref | ref | ref | ref | ref | ref |
| **Q2** | 2.670(1.118,6.378) | 0.027 | 2.342(0.970,5.657) | 0.059 | 2.586(1.070,6.250) | 0.035 |
| **Q3** | 3.868(1.783,8.392) | <0.001 | 3.893(1.771,8.558) | <0.001 | 4.152(1.819,9.478) | <0.001 |
| **Q4** | 3.058(1.424,6.568) | 0.004 | 3.107(1.234,7.824) | 0.016 | 3.271(1.214,8.810) | 0.019 |
| **P for trend** |  | 0.003 |  | 0.005 |  | 0.008 |
| **TRUNK FMR, every 0.1 point** | 1.151(1.045,1.269) | 0.004 | 1.255(1.095,1.438) | 0.001 | 1.260(1.083,1.466) | 0.003 |
| **TRUNK quartile** |  |  |  |  |  |  |
| **Q1** | ref | ref | ref | ref | ref | ref |
| **Q2** | 2.056(0.839,5.038) | 0.115 | 1.985(0.857,4.599) | 0.11 | 2.270(0.969,5.318) | 0.059 |
| **Q3** | 1.677(0.669,4.205) | 0.27 | 1.615(0.628,4.152) | 0.32 | 1.757(0.725,4.258) | 0.212 |
| **Q4** | 2.370(1.114,5.043) | 0.025 | 2.761(1.193,6.393) | 0.018 | 2.849(1.201,6.753) | 0.017 |
| **P for trend** |  | 0.023 |  | 0.022 |  | 0.034 |
| **TOTAL FMR, every 0.1 point** | 1.186(1.068,1.318) | 0.001 | 1.421(1.186,1.702) | <0.001 | 1.429(1.174,1.740) | <0.001 |
| **TOTAL quartile** |  |  |  |  |  |  |
| **Q1** | ref | ref | ref | ref | ref | ref |
| **Q2** | 4.428(1.840,10.656) | <0.001 | 3.114(1.240, 7.823) | 0.016 | 3.324(1.349, 8.190) | 0.009 |
| **Q3** | 3.541(1.495, 8.384) | 0.004 | 3.734(1.432, 9.739) | 0.007 | 3.931(1.462,10.569) | 0.007 |
| **Q4** | 4.189(1.900, 9.234) | <0.001 | 6.177(2.239,17.047) | <0.001 | 6.236(2.084,18.663) | 0.001 |
| **P for trend** |  | 0.004 |  | 0.002 |  | 0.005 |

Crude Model: No-adjust

Model 1: Age, Sex, Race, PIR, marital status, Educational Level

Model 2: Model 1, Smoke, Drinking, Physical Activity, Hypertension, CVD, Antihyperlipidemic Drugs, BMI, TC, TG and HDL

**Table S7** Association Between Fat-to-Muscle Ratio and Cardiovascular Disease Mortality in Diabetic Kidney Disease Patients: A Competing Risk Analysis.

| Variable | Sub-distribution HR | 95%CI | P value |
| --- | --- | --- | --- |
| **FMR.ARM** | 1.121 | 1.06-1.19 | 0.0002 |
| **FMR.LEG** | 1.155 | 1.06-1.25 | 0.0007 |
| **FMR.TRUNK** | 1.269 | 1.13-1.42 | <0.0001 |
| **FMR** | 2.074 | 2.06-2.09 | <0.0001 |

**Table S8.**Participant demographics and characteristics from the real-word cohort.

|  | Total | No-DKD | DKD | Pvalue |
| --- | --- | --- | --- | --- |
| n | 94 | 63 | 31 |  |
| HbA1c | 7.800(6.900,9.900) | 7.300 (6.800,9.400) | 9.400(7.450,10.425) | 0.004 |
| Age, years | 64.000(53.000,70.000) | 62.000(55.000,69.000) | 66.000(52.000,72.000) | 0.412 |
| BMI, kg/m^2^ | 24.629(22.188,27.162) | 23.967(20.400,26.379) | 25.219(24.021,27.766) | 0.033 |
| TG, mmol/L | 1.380(0.990,2.000) | 1.310(0.880,1.990) | 1.415(1.003,2.443) | 0.242 |
| TC, mmol/L | 4.251±0.140 | 4.265±0.174 | 4.225±0.237 | 0.730 |
| HDL, mmol/L | 1.097±0.038 | 1.104±0.042 | 1.084±0.078 | 0.965 |
| arm fat, g | 2088.500(1751.000,2557.000) | 2045.000(1730.000,2494.000) | 2184.000(1877.000,2581.000) | 0.205 |
| arm muscle mass, g | 4247.000(3421.750,5563.000) | 4408.000(3508.000,5770.000) | 3901.000(3295.000,4931.000) | 0.113 |
| leg fat, g | 4551.000(3663.500,5697.750) | 4455.000(3559.000,5683.000) | 4658.000(3899.000,5759.000) | 0.290 |
| leg muscle mass, g | 12100.500(10574.250,15354.000) | 12370.000(10695.000,15823.000) | 11608.000(10205.000,14503.000) | 0.200 |
| trunk fat, g | 12032.000(9132.000,15237.750) | 11197.000(7724.000,14211.000) | 13687.000(10655.000,15697.000) | 0.013 |
| trunk muscle mass, g | 20230.000(17505.750,24499.000) | 20290.000(17403.0000,24942.000) | 19749.000(17724.000,22324.000) | 0.775 |
| total fat, g | 19881.000(15492.750,23819.25) | 18917.000(13999.000,23157.000) | 22140.000(18454.000,22402.000) | 0.035 |
| total muscle mass, g | 39587.500(34764.250,49739.750) | 39987.000(34773.000,50569.000) | 38567.000(34674.0000,44718.000) | 0.517 |
| ARM-FMR | 0.534±0.024 | 0.496±0.028 | 0.606±0.043 | 0.450 |
| LEG-FMR | 0.401±0.019 | 0.377±0.021 | 0.446±0.036 | 0.510 |
| TRUNK-FMR | 0.605±0.025 | 0.563±0.031 | 0.687±0.039 | 0.022 |
| TOTAL-FMR | 0.505±0.019 | 0.471±0.023 | 0.571±0.034 | 0.016 |
| Sex |  |  |  | 0.611 |
| male | 49(52.100) | 34(54.000) | 15(48.400) |  |
| female | 45(47.900) | 29(46.000) | 16(51.600) |  |
| Smoking |  |  |  | 0.8951 |
| No | 72(76.600) | 48(76.200) | 24(77.400) |  |
| Yes | 22(23.400) | 15(23.800) | 7(22.600) |  |
| Hypertension |  |  |  | 0.3981 |
| No | 36(38.300) | 26(41.300) | 10(32.300) |  |
| Yes | 58(61.700) | 37(58.700) | 21(67.700) |  |
| CVD |  |  |  | 0.4931 |
| No | 82(87.200) | 56(88.900) | 26(83.900) |  |
| Yes | 12( 12.800) | 7( 11.100) | 5(16.100) |  |
| anti Hyperlipidemic |  |  |  | 0.137 |
| No | 17(18.100) | 14(22.200) | 3(9.700) |  |
| Yes | 77( 81.900) | 49( 82.400) | 28(90.300) |  |
| ACEI/ARB |  |  |  | 0.265 |
| No | 59(62.8) | 42(66.700) | 17(54.800) |  |
| Yes | 35(37.2) | 21(33.300) | 14(45.200) |  |

| **Table S9.** Univariate logistics analysis of the risk factors for the incident DKD in the real-world cohort. | | | | |
| --- | --- | --- | --- | --- |
| **Variable** | ***OR*** | ***P*** Value | **95%**CI Lower | **95%**CI Upper |
| Age | 1.012 | 0.5481 | 0.974 | 1.053 |
| Gender | 0.8 | 0.6108 | 0.335 | 1.895 |
| Smoking | 0.933 | 0.8948 | 0.32 | 2.534 |
| BMI | 1.072 | 0.157 | 0.975 | 1.185 |
| ACEI/ARB | 1.647 | 0.2665 | 0.68 | 3.991 |
| hypertension | 1.476 | 0.3993 | 0.606 | 3.751 |
| CVD | 1.538 | 0.4953 | 0.421 | 5.282 |
| Antihyperlipidemic Drugs | 2.667 | 0.1485 | 0.787 | 12.287 |
| HbA1c | 1.298 | 0.017 | 1.053 | 1.623 |
| TG | 1.436 | 0.0681 | 0.992 | 2.19 |
| TC | 1.059 | 0.7263 | 0.765 | 1.46 |
| HDL | 0.971 | 0.9643 | 0.256 | 3.484 |
| total.FMR | 23.132 | 0.0218 | 1.771 | 406.856 |
| trunk.FMR | 10.113 | 0.0274 | 1.404 | 90.209 |
| arms.FMR | 7.701 | 0.049 | 1.045 | 63.416 |
| legs.FMR | 13.52 | 0.0606 | 0.97 | 240.053 |
|  |  |  |  |  |

**Table S10**. Models comparison in the real-world cohort.

| Model | Variable | OR | 95% CI | P-Value | AUC | AIC | Sensitivity | Specificity |
| --- | --- | --- | --- | --- | --- | --- | --- | --- |
| Model 1 | Total FMR | 31.81 | (2.049 - 649.291) | 0.0169 | 0.731 | 111.53 | 0.71 | 0.73 |
|  | HbA1c | 1.344 | (1.074 - 1.718) | 0.0125 |  |  |  |  |
|  | TG | 1.41 | (0.956 - 2.169) | 0.0907 |  |  |  |  |
| **Model 2** | **Trunk FMR** | **12.029** | **(1.431 - 121.317)** | **0.0260** | **0.735** | **112.42** | **0.71** | **0.714** |
|  | HbA1c | 1.345 | (1.074 - 1.720) | 0.0125 |  |  |  |  |
|  | TG | 1.375 | (0.929 - 2.113) | 0.1195 |  |  |  |  |
| Model 3 | Arms FMR | 8.934 | (1.076 - 81.337) | 0.0448 | 0.728 | 113.58 | 0.742 | 0.746 |
|  | HbA1c | 1.309 | (1.053 - 1.653) | 0.0180 |  |  |  |  |
|  | TG | 1.449 | (0.988 - 2.232) | 0.0676 |  |  |  |  |
| Model 4 | Legs FMR | 25.7 | (1.532 - 567.784) | 0.0287 | 0.725 | 112.59 | 0.677 | 0.778 |
|  | HbA1c | 1.34 | (1.074 - 1.704) | 0.0121 |  |  |  |  |
|  | TG | 1.489 | (1.010 - 2.301) | 0.0523 |  |  |  |  |
| Model 5 | HbA1c | 1.299 | (1.050 - 1.630) | 0.0186 | 0.699 | 115.69 | 0.742 | 0.698 |
|  | TG | 1.439 | (0.984 - 2.200) | 0.0707 |  |  |  |  |

Model 1: Tatal FMR, HbA1c, TG

Model 2: Trunk FMR, HbA1c, TG

Model 3: Arms FMR, HbA1c, TG

Model 4: Legs FMR, HbA1c, TG

Model 5: no FMR, HbA1c, TG


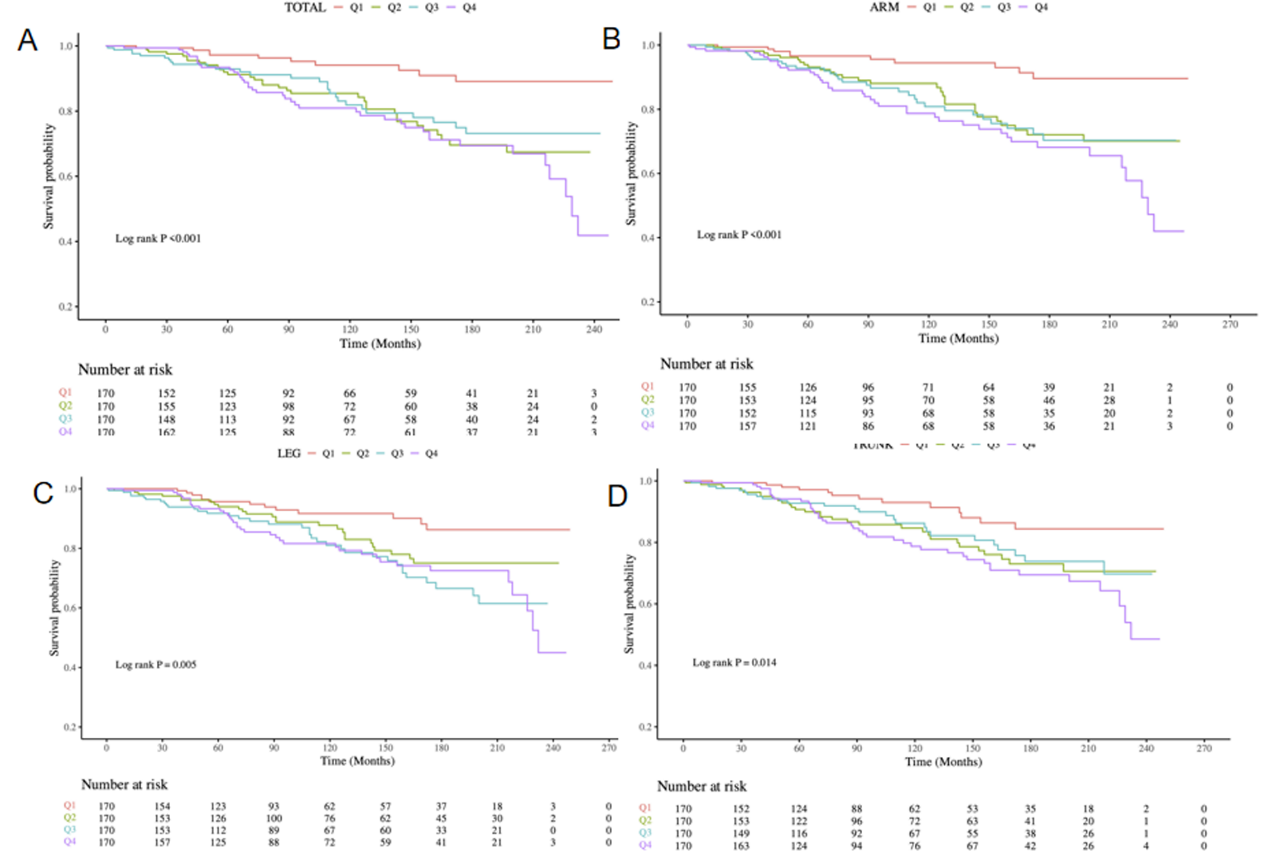


**Fig.S1** Kaplan-Meier curves displayed for CVD mortality according to the quartiles of FMR.


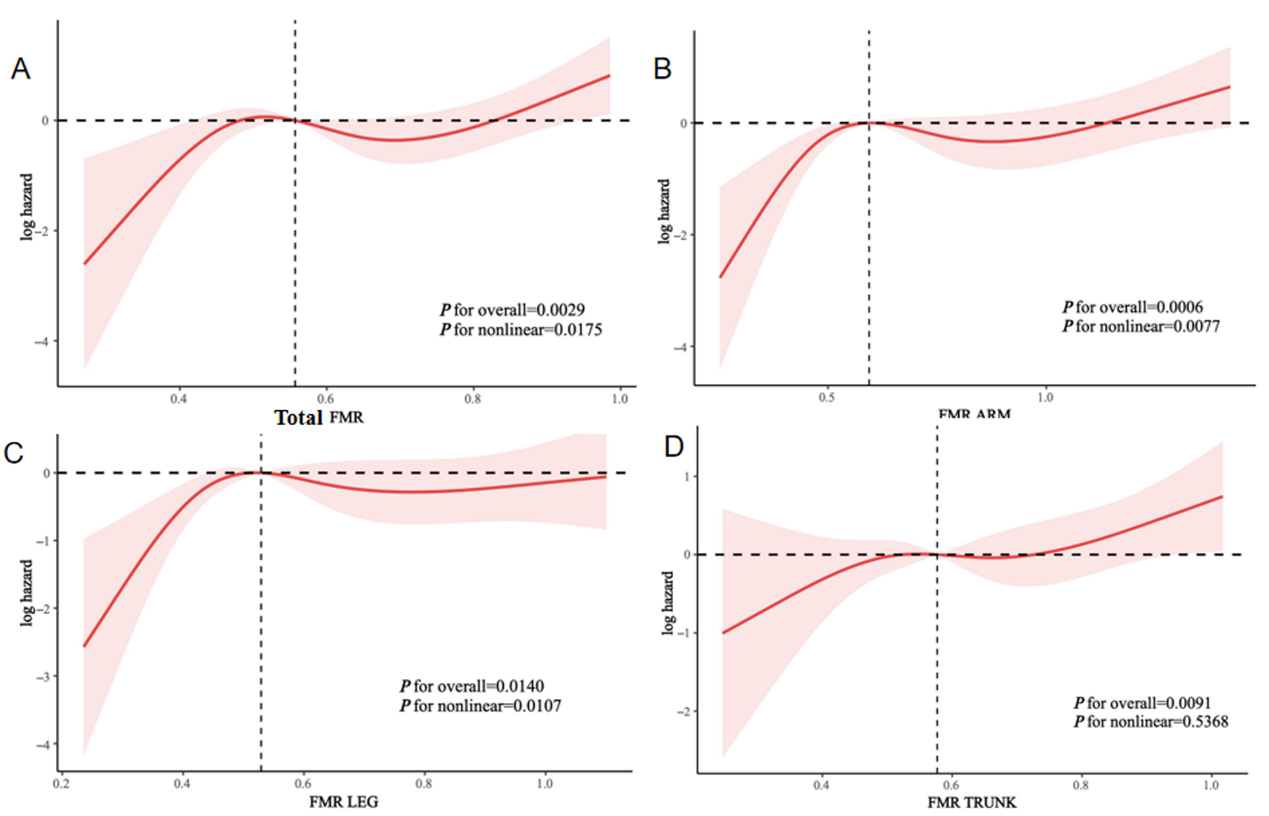


**Fig.S2** Non-linear associations between different FMRs and CVD mortality.


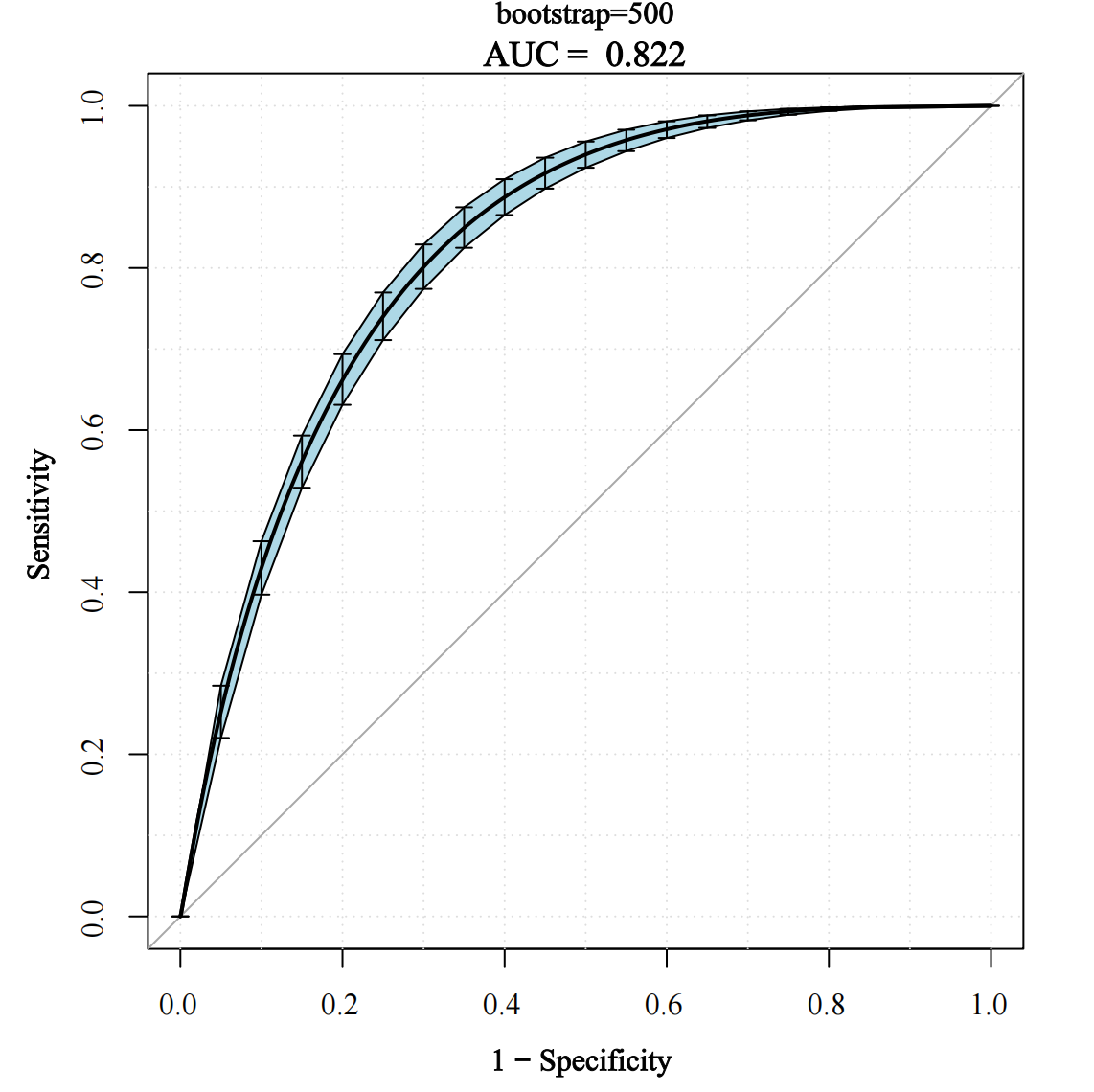


**Fig.S3** Bootstrap Validation of the Trunk-FMR Model ROC Curve (500 Replicates).
